# The contribution of dormant origins to genome stability: From cell biology to human genetics

**DOI:** 10.1016/j.dnarep.2014.03.012

**Published:** 2014-07

**Authors:** Robert C. Alver, Gaganmeet Singh Chadha, J. Julian Blow

**Affiliations:** Centre for Gene Regulation & Expression, College of Life Sciences, University of Dundee, Dow Street, Dundee DD1 5EH, UK

**Keywords:** MCM2–7, Origin licensing, Replication origins, Dormant origins, Meier–Gorlin, Pre-RC

## Abstract

The ability of a eukaryotic cell to precisely and accurately replicate its DNA is crucial to maintain genome stability. Here we describe our current understanding of the process by which origins are licensed for DNA replication and review recent work suggesting that fork stalling has exerted a strong selective pressure on the positioning of licensed origins. In light of this, we discuss the complex and disparate phenotypes observed in mouse models and humans patients that arise due to defects in replication licensing proteins.

## 1 Introduction

**Fig. 1:**
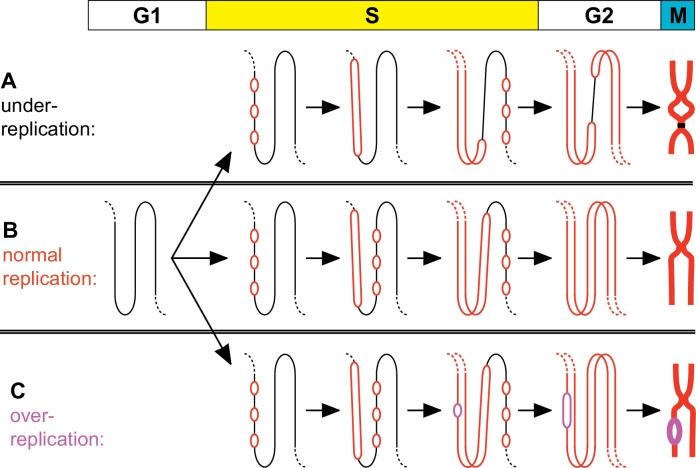
Ensuring precise chromosome replication. A small segment of chromosomal DNA is shown, consisting of three domains each replicated from three replication origins. The domain is shown at different stages of the cell cycle: G1, early-, mid- and late-S phase and G2; a whole chromosome containing the chromosomal segment is shown in mitosis (‘M’). (A) The DNA is under-replicated as a consequence of origins in the middle cluster failing to fire. As sister chromatids are separated during anaphase, the chromosome is likely to be broken near the unreplicated section. (B) Origins are correctly used and chromosomal DNA is successfully duplicated. (C) One of the origins fires for a second time in S phase. The local duplication of DNA in the vicinity of the over-firing origin represents an irreversible genetic change and might be resolved to form a tandem duplication. Reproduced from [11].

Because of the very large size of eukaryotic chromosomes, they need to be replicated by many hundreds or thousands of replication forks which are initiated from replication origins spaced throughout the genome. The use of multiple replication origins not only ensures timely completion of genome duplication, but also allows cells to replicate different regions of the genome at different stages of S phase (the ‘replication timing programme’)—this may help cells assemble nascent DNA into different chromatin or transcriptional states. However, the use of multiple replication origins makes it more difficult to ensure that the entire genome is precisely duplicated during each S phase, with no sections left unreplicated and no sections replicated more than once (under- and over-replication, Fig. 1). Cells resolve these challenges and preserve genome integrity by dividing the whole process of replication initiation into two distinct non-overlapping steps: origin ‘licensing’ which occurs in late mitosis and G1, and origin ‘firing’ which occurs during S phase [2,9,33].

From late mitosis through G1 phase, replication origins are licensed for use in the upcoming S-phase by loading double hexamers of MCM2–7 (mini chromosome maintenance) proteins onto DNA [25,28,33,63]. During S phase, two S-phase kinases Cdc7 and CDKs promote the binding of Cdc45 and the GINS complex to some of the MCM2–7 hexamers at licensed origins. This forms a functional CMG (CDC45, MCM2–7, GINS) helicase which powers the progression of the replication fork [41,54]. Origin firing and consequent movement of the CMG away from the origin reverts it back to an unlicensed state. Additionally, as active replication forks encounter MCM2–7 hexamers at unfired origins, the inactive MCM2–7 complexes are removed from the DNA. This combination of features prevents the re-replication of chromosomal DNA.

In order to prevent DNA re-replication, it is critical that any further origin licensing ceases at the onset of S-phase [2,9]. As a consequence, if problems occur during S phase – such as the stalling or disassembly of replication forks – the cell cannot alleviate the problem by licensing new origins. Cells therefore license many more origins than are normally used, with many origins remaining ‘dormant’ to provide a backup in case of problems during S phase [11].

Here we will discuss the recent evidence from yeast showing that these pressures have had a major influence on the distribution of replication origins. We will describe how activation of otherwise dormant replication origins provides an important defence against many potential genotoxic stresses. We will review work on mouse and human mutant genes that are involved in origin licensing and discuss how these mutations might cause the observed cellular and developmental defects.

## 2 Origin licensing

Origin licensing in late mitosis and G1 occurs in a series of biochemical steps that result in the clamping of two MCM2–7 hexamers in an antiparallel conformation around double stranded DNA [25,28,33,63]. This assembly is driven by three essential factors which together with MCM2–7 form the ‘pre-replicative complex’ (pre-RC): ORC (origin recognition complex), CDC6 and CDT1. ORC consists of 6 subunits, ORC1 to ORC6, though in some cell types ORC6 is absent from the complex. ORC binds to origin DNA, and then promotes the association of CDC6, which then with the help of CDT1 recruits two hexamers of MCM2–7 [26,27]. Once a double hexamer is loaded, it remains stably associated with the DNA until the DNA is replicated [44,70,72].

In contrast to MCM2–7, the other pre-RC components only associate transiently with the DNA. Photobleaching studies of GFP-tagged ORC subunits in Chinese Hamster Ovary cells and in *Caenorhabditis elegans*, show that ORC turnover on DNA typically occurs in a few seconds [53,70]. A similar dynamic association of CDC6 and CDT1 with DNA has also been reported [83,70]. Interestingly, in both *Xenopus laevis* and *C. elegans* early embryos, the loading of MCM2–7 onto DNA appears to promote the destabilization of ORC, CDC6 and CDT1 [59,70]. This is consistent with observations made in the reconstituted *Saccharomyces cerevisiae* system, where ORC and CDC6 are probably ejected from the origin in a mechanism involving ATP hydrolysis [13,27,78]. Since MCM2–7 double hexamers are loaded in a considerable excess over the amount of ORC, it has been proposed that the destabilization of the ORC, CDC6 and CDT1 complex that occurs when origins are licensed could provide a mechanism for distributing replication origins along chromosomal DNA [70]. This is consistent with studies showing that there is a significant excess of MCM2–7 double-hexamers loaded onto DNA when compared to the amount of ORC [11,22,24,52,81].

In every cell type examined so far, there is also a 3- to 20-fold excess of MCM2–7 double hexamers loaded onto DNA over the number of origins that are actually used in any individual S phase [11,39,81]. One explanation for this ‘MCM paradox’ is that only a fraction of licensed origins are actually used in any given S phase, with the majority remaining dormant. The existence of dormant replication origins is clearly revealed under conditions of replication stress: if replication forks stall or their progression is impeded, dormant origins are activated and this is important so that the entire genome can be completely replicated (see Section 4 on ‘dormant origins’) [1,11,30,82]. In addition, it is possible that more than one MCM2–7 double hexamer might be loaded at certain origins. Having several MCM2–7 double hexamers at one site could increase the probability of the origin firing, which provides various theoretical advantages to organising S phase [42,65,85]. However, to date no direct evidence has been reported for such a hyperloading of MCM2–7 at individual origins.

## 3 Origin distribution

For all functional purposes, a DNA sequence gains the *potential* to act as an origin by being loaded with MCM2–7 double hexamers (i.e. by becoming licensed) whilst these licensed sites *actually* become replication origins in a cell only when the MCM2–7 hexamers are transformed into an active CMG helicase. The features that specify metazoan replication origins have been debated for a long while, and much still remains unclear. In *S. cerevisiae*, ORC binds to an A/T-rich consensus sequence (ACS). Possession of this consensus sequence is not sufficient to predict the existence of a functional DNA replication origin. In fact, out of the 12,000 ACS sites identified only 400 are functional [58]. The location of the ACS within an extended nucleosome-free region may contribute to it becoming a functional origin [23]. There is no analogous ACS in the distantly related yeast *Schizosaccharomyces pombe*, though the position of replication origins correlates highly with AT-rich and poly-A DNA [84]. These features probably contribute to origin specification in at least two ways: first, *S. pombe* ORC shows a strong preference for binding AT-rich DNA, and second, AT-richness may help create nucleosome-free regions which promote origin activity [84]. In metazoan cells, origin specification is even less well understood than in yeasts, though recent deep sequencing studies have highlighted the possible importance of GC-rich sequence elements [5,14,16].

What is the significance of positioning replication origins at particular places on chromosomal DNA? It is likely that certain regions are unfavourable for locating replication origins, such as within complex promoters or in the middle of highly transcribed genes. But these provide very limited constraints on where origins might actually be placed. If origins were placed at random sites on the genome, this would result in some adjacent replication origins being very far apart. There are two obvious negative consequences of having a few widely-spaced replication origins. Firstly, this in principle sets the minimum time for the entire genome to be replicated as the time taken for the two forks initiated from the most widely-spaced pair of origins to traverse the gap between them. This is likely to be an important limitation for organisms such as early embryos which have a very short S phase. But for somatic cells, with a much longer S phase driven by a replication timing programme and active checkpoint responses to delay entry into S phase, this might not pose a potential threat. A second negative consequence of having large inter-origin gaps arises from problems that occur if replication forks irreversibly stall, for example, after encountering damaged or chemically modified DNA [57]. Because licensing only occurs before the onset of S phase, no new origins can be licensed to rescue these stalled forks. This is potentially an important problem for all cells.

**Fig. 2:**
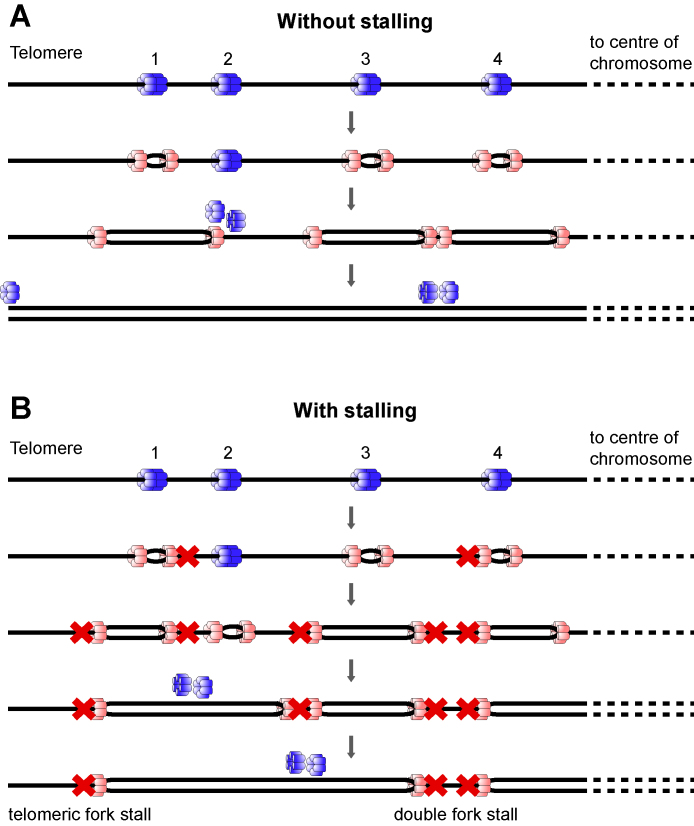
Cartoon of replication origins near the end of a chromosome. DNA is denoted as a single black line, with a telomere (chromosome end) to the left. Prior to S phase entry, origins are licensed by binding a double hexamer of MCM2–7 proteins (blue). As an origin fires, both MCM2–7 single hexamers are converted into an active CMG helicase (pink). (A) Forks initiate at origins 1, 3 and 4. If an active fork passively replicates an inactive origin, the MCM2–7 at the inactive origin is displaced making the origin dormant (origin 2) for that particular cell cycle. (B) In case of irreversible fork stalling (denoted by a red cross) otherwise dormant origins can be activated (origin 2) to ensure complete replication of the DNA. If both of the converging forks stall (‘double fork stall’) without a dormant origin existing between them (as occurs at forks converging between origins 3 and 4), replication of the intervening DNA is compromised. If the single fork heading towards the telomere (the fork move left from origin 1) irreversibly stalls and there is no telomere-distal origin, (‘telomeric fork stall’), then this single stall event can also compromise full replication of the genome. Reproduced from [57].

When forks encounter barriers to their movement, such as might be created by DNA damage or proteins tightly bound to DNA, this can lead to an irreversible fork arrest. Some protection against fork stalling arises from the fact that each origin initiates a pair of bi-directional forks, so that if one of the converging forks stalls, the other fork can compensate and replicate all of the intervening DNA (Fig. 2A). However, if both converging forks stall (a ‘double fork stall’), replication of the intervening DNA is compromised (Fig. 2B). A new origin cannot be licensed between the two stalled forks, because new origin licensing is prohibited once S phase has begun in order to prevent re-replication of chromosomal segments [2,9,12,57]. It should also be noted that there is a particular problem with DNA at the end of a chromosome, which can only be replicated by forks coming from the body of the chromosome. Replication can fail at chromosome ends if a single replication fork stalls in telomeric or subtelomeric DNA and there is no other licensed origin distal to the stalled fork (Fig. 2B, ‘telomeric fork stall’).

The theoretical estimations of replication origin spacing required to minimize ‘double fork stalls’ or ‘telomeric fork stalls’ has been compared with the actual origin positions in five different yeast species [57]. The probability of a double fork stall increases as the square of the distance between two adjacent origins, so double fork stalls are proportionately more likely to occur between distantly spaced origins. Large inter-origin distances should therefore be avoided, and in all 5 yeasts examined (*S. cerevisiae*, *S. pombe*, *Kluyveromyces lactis*, *Lachancea kluyveri* and *Lachancea waltii*) this is clearly the case. Indeed, previous work [75] had shown that deletion of five origins in *S. cerevisiae*, creating a large inter-origin distance of 160 kb (close to the expected value of the largest inter-origin distance if origins were randomly distributed), resulted in an increased chromosome loss rate exactly in line with the increased probability of double fork stalls [57]. In order to globally minimize large gaps and the probability of double fork stalls, it is also optimal to position replication origins at regularly spaced intervals across the genome. Consistent with this, replication origins in all 5 yeasts showed a significant degree of regularity in their spacing [57]. Chromosomal ends are in a precarious situation since there is no converging fork that can rescue fork stalling from the most distal fork found near the chromosome end. This fact allows the prediction that origins should be located very close to the end of chromosomes. Indeed, this is the case for all 16 *S. cerevisiae* chromosomes, with the average origin distance from the chromosome ends being ∼50 times smaller than the average inter-origin distance in the body of chromosomes. Taken together, all these considerations suggest that replisome stall events have strongly shaped the distribution of replication origins in yeasts [57].

The probability that a double fork stall occurs somewhere in the genome is dependent on three main factors: (i) the genome size, (ii) the distance between licensed origins and iii) the distance that replication forks would be expected to travel before they irreversibly stall. Newman et al. [57] used a range of published data to estimate a median stall distance of ∼10 Mbp in unstressed cells. Interestingly, this is about the size of the genomes of the five yeasts studied, and predicts that double fork stalls will be rare events that occur at a frequency similar to the natural chromosome loss rate. But for metazoans with much larger genome sizes than yeasts, the model predicts that double fork stalls become highly likely and might be expected to occur in ∼50% of all S phases in a typical human somatic cell.

Chromosome fragile sites in metazoan cells may represent a similar challenge to the large inter-origin gaps studied in yeasts. Fragile sites are chromosomal regions where there is a high frequency of chromosomal breaks and rearrangements due to failures in the process of DNA replication. The resulting DNA breaks at these sites may play an important role in tumourigenesis [20,60,79]. DNA fibre analysis of replication at fragile sites revealed paucity of active replication origins in these regions [20]. Replication defects at fragile sites may be a consequence of a low density of licensed origins or it may reflect inefficient or delayed initiation of replication forks.

## 4 Regulation of dormant origins

Not all licensed origins actually fire during a given S phase, but instead remain dormant and are passively replicated by forks emanating from flanking origins. When replication fork progression is inhibited, for example as a consequence of reduced dNTP supply or due to forks encountering DNA damage, some origins which otherwise would have remained dormant are activated. Under normal (unstressed) conditions, MCM levels can be reduced approximately 3 to 10-fold without any clear effect on the kinetics of S phase progression or the distribution of active origins. However, when exposed to replicative stresses, cells with lowered MCM levels have a reduced number of dormant origins, leading to a reduced replication rate, greater signs of DNA damage and checkpoint activation, and decreased levels of cell survival [11,30,40,82].

Dormant origins must be regulated in such a way that they are only active when needed, but how is this achieved? DNA fibre analysis of replication in metazoans shows that ‘clusters’ of 2 to 10 adjacent replication origins, each spanning a region of 0.5–1 Mbp, are activated near synchronously in S phase. In addition, experiments utilizing light microscopy have established that most DNA replication occurs in discrete ‘foci’ or ‘factories’, sub-nuclear structures which are enriched for active replication proteins [32]. Each factory is estimated to contain 4–20 replication forks and is likely to contain replication forks initiated from a single cluster of origins [32]. Different regions of the genome are replicated at different stages of S phase and in a predictable, evolutionarily conserved and cell type specific manner [64,66]; this defines the replication-timing program. However, which particular origins actually fire in a given cell cycle and which origins remain dormant appears to be stochastic [1,21,31,47,65]. This observed stochasticity could be due to the intrinsic inefficiency of origin firing which itself may be a mechanism for regulating dormant origins. Because fork slowing reduces the rate at which dormant origins are passively replicated by adjacent origins, the probability of a dormant origin becoming active increases when replication forks slow [10]. In this way, dormant origin activation occurring in response to fork stalling is a simple consequence of origin activation being stochastic. This simple system obviates the need for additional regulatory pathways to activate dormant origins when the cell undergoes replication stress. However it is also likely that dormant origins are, at least in part, regulated by active mechanisms.

The protection against double-fork stalling that is achieved by increasing the total number of licensed origins does not depend on whether these origins are efficient or whether they normally remain dormant [10]. When cells are forced to fire an excessive number of replication origins, the demand for replication factors increases dramatically, and it is potentially disastrous to initiate replication using a replisome missing critical components that have become limiting. Indeed, it has been observed that unrestrained origin firing causes exhaustion of the pool of RPA, which in turn causes exposure of single stranded DNA and subsequent DNA strand breakage [77]. Therefore, it is necessary to direct dormant origin firing specifically in the vicinity of the replication stress (locally) whilst inhibiting origin firing where replication has not yet initiated (globally). Two key factors have been identified that mediate this specific effect: the checkpoint kinase ATR and its downstream effector kinase Chk1.

When cells are challenged with replication stress, single stranded DNA coated with RPA becomes exposed due to a decoupling of the helicase and polymerase activities at the replication fork: this is the substrate for ATR recruitment and activation, which subsequently activates Chk1 kinase. ATR and Chk1 are known to globally inhibit the rate of replication initiation. At low levels of activity however, Chk1 preferentially inhibits the activation of new replication factories rather than the initiation of dormant origins within currently active factories [29]. The mechanism by which this happens is unclear, but one possibility is that ATR and Chk1 modestly reduce S phase Cdk levels, which has been shown to reduce the level of active replication factories [76]. At the same time, the slowing of replication forks within active factories stimulates dormant origin firing, either by the ‘passive’ mechanism described above, or possibly by some other active mechanism. Active ATR is expected to be enriched at stalled or slowed replication forks, and would be in a good position to stimulate initiation of nearby unfired origins. Although ATR is known to phosphorylate the MCM2–7 proteins [19], there is currently no direct evidence that this promotes initiation.

**Fig. 3:**
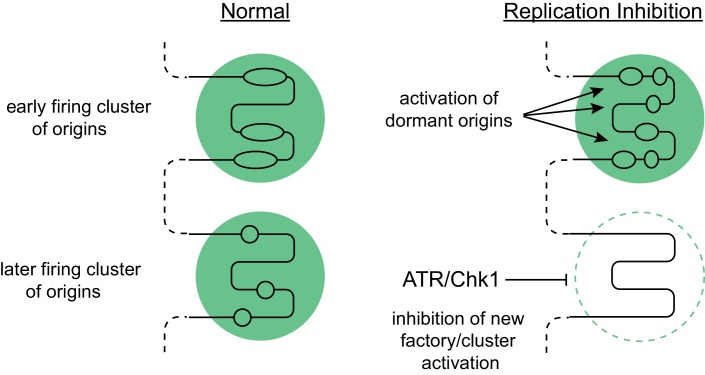
Model for how cells respond to low levels of replicative stress. Two adjacent clusters of origins (factories bounded by green circles) are shown on a single piece of DNA (black lines). Under normal circumstances (left), the upper factory is activated slightly earlier than the factory below, and each initiates three origins. Under low levels of replicative stress (right), replication forks are inhibited in the earlier replicating cluster, which promotes the firing of dormant origins as a direct consequence of stochastic origin firing. Replicative stress activates DNA damage checkpoint kinases, which preferentially inhibit the activation of the unfired later clusters/new factories. Reproduced from [29].

These two responses to replication fork inhibition – local activation of dormant origins and global suppression of factory activation – work together to direct new initiation events towards regions of the genome currently experiencing replicative problems whilst at the same time limiting overall origin activation which otherwise might lead to depletion of key proteins such as RPA [11,29,77]. This combined response minimizes the deleterious consequences of fork stalling and prevents similar problems from arising in unreplicated regions of the genome (Fig. 3).

## 5 The consequences of limited licensing

**Fig. 4:**
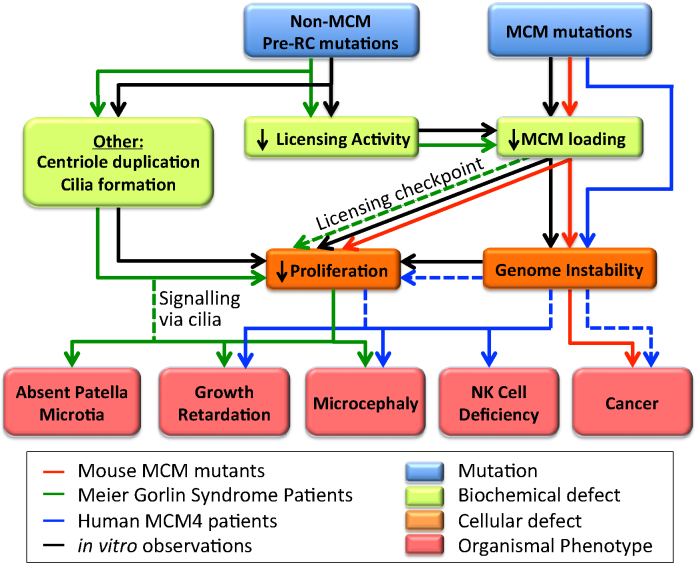
The consequences of limited licensing. Mutations of pre-RC components have been organized into two groups, MCMs, and non-MCMs (blue rectangles). The biochemical consequences for these mutations are then organized into three groups: MCM loading, decreased licensing activity, and ‘other’ (green rectangles). In turn, these biochemical defects lead to more general cellular defects, decreased proliferation and genome instability (orange rectangles). Subsequently, these cellular defects manifest as a phenotype observed at the organismal level (red rectangles). The arrows drawn from mutation to biochemical defect, to cellular defect, to phenotype are colour coded in reference to the type of experimental system in which the observations have been made (red: mouse models harbouring MCM hypomorphic alleles; green: Meier Gorlin patients; blue: human MCM4 patients; black: in vitro experimental systems). Lines are dashed where assumptions are made in line with observations, but it is unclear if a direct cause and consequence can be conclusively drawn.

Recent studies have determined the biochemical, cellular and phenotypic consequences of limited licensing in mice and human systems. Fig. 4 outlines the different consequences of limiting MCM content (right) or of limiting other pre-RC proteins (ORC/CDC6; left) in either whole animals (coloured lines) or in vitro experiments (black lines). The consequences of limiting cellular MCM content in whole organisms are, in general, consistent with in vitro work, showing evidence of increased DNA damage and genome instability. However, the situation appears more complex when other pre-RC components become limiting. In particular, the human genetic disorder Meier–Gorlin syndrome (MGS), associated with defective non-MCM pre-RC proteins is characterized by a number of unexpected developmental defects.

In mice harbouring the hypomorphic allele MCM4^Chaos3/Chaos3^, the mutant MCM4 protein is destabilized and only approximately half the wild type levels of functional MCM2–7 heterohexamers are loaded onto DNA [43,68]. This reduction limits the number of dormant origins that are activated in response to replicative stresses and results in genome instability. These cellular defects are not strictly dependent on the individual hypomorphic MCM allele used, as a mouse model utilizing an MCM2^IRES-CreERT2/IRES-CreERT2^ allele, which resulted in a reduction in total protein level to roughly 1/3 the wild type amount, exhibited similar defects in dormant origin usage and genome instability [45,62]. A third hypomorphic allele, MCM4 D573H, acts in a dominant way to make a non-functional helicase whilst not affecting the stability of the mutant protein itself [3]. Cells bearing any of these three mutations show evidence of increased DNA damage and have unstable genomes. Consistent with their observed genome instability, these mouse models are highly cancer prone. In addition, there is some evidence that MCM hypomorphic mice have severe deficiencies in the proliferative cell compartments of a variety of tissues, potentially due to a depletion of stem cells [62].

A set of human patients that present with natural killer cell deficiency, growth retardation, adrenal insufficiency, and genome instability were recently shown to harbour a mutation resulting in expression of a truncated form of MCM4 [15,34,38]. Though this truncated form does not seem to affect loading of the helicase onto DNA, cells from these patients exhibit increased levels of chromosome breakage as well as a defective cell cycle. Human tissue culture experiments in U2OS and HeLa cell lines utilizing siRNA mediated knockdown of individual MCM subunits corroborate the biochemical and cellular defects observed in the mouse models [30,40]. These data combined with the results from similar experiments in *Danio rerio* [67], *S. pombe* [48], and *C. elegans* [82] all lead to the conclusion that there is a threshold number of properly functioning, licensed origins that needs to be maintained in order to protect the cell from chromosomal instability and carcinogenesis.

Because of the inherent risk of trying to replicate the genome with too few licensed origins, it would make sense if cells had a way to ensure that a sufficient number of origins have been licensed in late G1 before the licensing system is shut down in preparation for entry into S phase. Consistent with this idea, it has been shown that certain metazoan cell lines possess a “licensing checkpoint” that prevents G1 cells from entering S phase before the licensing system is inhibited [49,51,56,69,74]. This checkpoint arrests cells in late G1 prior to full Cdk activation and consequent phosphorylation of the retinoblastoma protein Rb, at a cell cycle stage where further origin licensing should be possible. The precise molecular pathway underpinning the licensing checkpoint is currently unclear, but appears to involve p53 and converges on down-regulating G1/S Cdk2 activity. The licensing checkpoint is defective in many cancer cell lines, possibly because of its dependency on the p53-Rb control system [49,56,69]. When licensing is inhibited in cells that do not have a robust licensing checkpoint, cells progress into an S phase they cannot complete, activate a DNA damage response and ultimately die.

Another cellular defect that has been observed in mouse MCM hypomorphs is an overall decrease in cellular proliferation, possibly associated with a depletion of stem cell populations [45,62]. It is difficult to discern precisely why the proliferation rate is decreased in these experiments: these cells all exhibit genome instability, which means that the DNA damage response will be engaged and is expected to slow cell cycle progression. Additionally, if the licensing checkpoint is activated this would delay progression into S phase and inhibit cellular proliferation.

In contrast to the phenotype of MCM hypomorphic mice, human patients with Meier Gorlin syndrome (MGS), a rare disorder linked to defective non-MCM pre-RC proteins (ORC1, ORC4, ORC6, CDT1, and CDC6) is characterized by primordial dwarfism, mild to severe microcephaly, and hypoplasia of the ear and patella [6,7,35]. Some of these defects have been suggested to result from cell-type specific proliferation defects during development. The mutations found in MGS patients result in a spectrum of biochemical and cellular phenotypes that partially overlap with the effect of MCM mutations, including impaired licensing, altered S phase progression and proliferation defects. Notably absent from this list of phenotypes is chromosomal instability or an increased predisposition to cancer. It is possible that in most cell types, the degree of licensing inhibition in MGS patients is slight enough to predominantly impact on development via activation of the licensing checkpoint, resulting in a reduced number of cells in certain key cell types such as neurons. However, at least under certain conditions, MGS mutations can cause fairly significant reduction in origin licensing [7,8]. It is also possible that some MGS individuals might have an increased risk of cancer, but because MGS is such a rare disorder this has not become apparent in the clinical record.

Another possible explanation for the difference between the phenotype of MGS and MCM hypomorphs is that it stems from functions of the pre-RC proteins beyond their canonical role in origin licensing. A large number of studies have shown that non-MCM pre-RC proteins are involved in mitotic events independent of their role in origin licensing. The moonlighting of pre-RC proteins in mitotic functions may reflect a closer connection between replication origins and chromosome segregation in the ancestral eukaryotic cell. ORC1, the largest ORC subunit is important for the regulation of centrosome duplication [36,46], whilst the smallest ORC subunit ORC6 has a role in cytokinesis [4,17,61]. ORC1 mutations found in MGS patient cells promote centrosome reduplication, most likely by affecting the ability of ORC1 to restrain centrosome duplication via inhibition of Cyclin E-CDK2 kinase [37]. Similarly, CDT1 promotes microtubule attachment to kinetochores [80], whilst the CDT1 inhibitor, geminin, is involved in preventing centrosome over-duplication [73]. In addition, cilia develop from centrosomes/centrioles and ORC1, ORC4, ORC6, CDC6, and CDT1 have all been implicated in cilia formation [71]. Given that several signalling pathways, such as Hedgehog signalling, depend on cilia, this provides another route by which MGS mutations could reduce cellular proliferation rates without contributing to genetic instability. CDC6 also has a non-licensing role in regulating the checkpoint kinase, ATR [18,55,59]. This may be relevant to the MGS phenotype as ATR mutations are implicated in Seckel syndrome, which has several overlapping features with MGS, including microcephaly [71].

## 6 Future perspectives

The organismal phenotypes of mutations in different pre-RC components seem to reflect different contributions of their licensing and non-licensing roles. The MCM proteins predominantly function in DNA replication, and mutants therefore have consequences resulting in defective proliferation, genome instability and cancer. The other pre-RC proteins have a range of additional functions, primarily centred around the centrosome, which can inhibit cellular proliferation without significantly promoting genetic instability. Additional signalling defects mediated by defective formation of cilia may also play a role. These additional roles could explain some of the more unexpected features of MGS (Fig. 4).

MGS therefore potentially provides an instructive disease for understanding how mutations in proteins with promiscuous roles can generate complex phenotypes. One way this can be exploited is to distinguish mutations that cause a phenotype by generally lowering protein function (as appears to be the case with the MCM4-Chaos and MCM2-IRES mutants) from mutations that might selectively affect one particular function (such as appears to be the case with the ORC1 MGS mutation). Deeper understanding of how MGS mutations cause the disease phenotypes will illuminate how their respective genes function in the context of interlocking regulatory systems.

The organismal phenotypes caused by mutations in the licensing system also highlights our limited understanding of what happens to cells when the DNA replication programme is compromised. It is currently unclear what the threshold values are for the number of licensed origins that will trigger the ‘licensing checkpoint’ and whether this varies between cell types. Understanding this will have implications for the development of novel anticancer targets that target the initiation of DNA replication [12]. The prediction that in somatic mammalian cells there is a high probability of the occurrence of double fork stalls suggests that these cells will have evolved mechanisms for effectively dealing with the consequences. Some novel mechanisms have been proposed that could deal with this sort of problem [50,86] and it will be interesting to see how these pathways are affected when the activity of the licensing system is compromised.

## Conflict of interest statement

We declare no conflict of interest.
